# Repositioning synthetic glucocorticoids in psychiatric disease associated with neural autoantibodies: a narrative review

**DOI:** 10.1007/s00702-022-02578-2

**Published:** 2022-12-28

**Authors:** Niels Hansen, Alexandra Neyazi, Daniel Lüdecke, Alkomiet Hasan, Jens Wiltfang, Berend Malchow, the CAP (Cerebrospinal Fluid Analysis in Psychiatry) Consortium

**Affiliations:** 1 Department of Psychiatry and Psychotherapy, University Medical Center Göttingen, Von-Siebold-Str. 5, 37075 Göttingen, Germany; 2 Department of Psychiatry and Psychotherapy, Otto-Von-Guericke-University Magdeburg, Magdeburg, Germany; 3 Department of Psychiatry and Psychotherapy, University Hospital Hamburg Eppendorf, Hamburg, Germany; 4 Department of Psychiatry, Psychotherapy and Psychosomatics, Medical Faculty, University of Augsburg, 86156 Augsburg, Germany; 5 German Center for Neurodegenerative Diseases (DZNE), Von-Siebold-Str. 3a, 37075 Goettingen, Germany; 6 Neurosciences and Signaling Group, Institute of Biomedicine (iBiMED), Department of Medical Sciences, University of Aveiro, Aveiro, Portugal

**Keywords:** Drug repositioning, Immunotherapy, Synthetic glucocorticoids, Psychiatry, Autoantibody

## Abstract

Synthetic glucocorticoids (sGCs) are a well-investigated and standard drug therapy for disorders associated with CNS inflammation. Less is known about treating psychiatric disorders associated with neural autoantibodies. Our aim is to elucidate the repositioning of sGCs in psychiatric diseases that co-exist with neural autoantibodies. We used PubMed to identify articles for this narrative review. To our knowledge, no randomized, placebo-controlled trials have yet been conducted on applying sGC to treat neural autoantibody-associated psychiatric disorders. We describe initial results of cohort studies and single cases or case series often associated with autoantibodies against membrane-surface antigens demonstrating a largely beneficial response to sGCs either as monotherapy or polytherapy together with other immunosuppressive agents. However, sGCs may be less efficient in patients with psychiatric diseases associated with autoantibodies directed against intracellular antigens. These results reveal potential benefits of the novel usage of sGCs for the indication of neural autoantibody-associated psychiatric disease. Further large-scale randomized, placebo-controlled trials are needed to discover whether sGCs are safe, well tolerated, and beneficial in subgroups of neural autoantibody-associated psychiatric diseases.

## Introduction

The term drug repurposing refers to employing old drugs for a new purpose, as for another disease category. Two additional subconditions of drug repurposing beyond its superordinate concept are distinguished, namely, drug repositioning and drug reformulation. Drug repositioning means employing an old formula for a new indication, whereas drug reformulation requires either the application of a novel dosage or a novel route, or even both. In our review, we focus on the drug repositioning of steroids in psychiatric disease limited to those patients presenting serum and/or CSF neural autoantibodies. In our review article, we refer only to synthetic glucocorticoids (sGC, i.e., methylprednisolone or prednisone) as they are currently applied in treating diseases involving CNS inflammation. sGCs orchestrate various cellular functions, such as inflammation (Rhen and Cidlowski 2005) with mainly anti-inflammatory effects, as is evident in cell cultures (Ryan et al. 2020), but also proinflammatory effects appear in rodent models (e.g., Spiga et al. 2020). sGCs are drugs for immunosuppression in neuropsychiatric disorders, such as autoinflammatory brain conditions termed autoimmune encephalitis encompassing seizures, psychiatric features, or memory disturbances along with prominent, but not exclusive affection of the temporal lobe (Graus et al. 2016; Abboud et al. 2021a, b) and autoimmune epilepsy referring to a chronic condition that predisposes to seizures on an autoimmune basis (Bhatia and Schmitt 2018). A recent overview depicted the diverse and often overlapping psychopathology detected in all patients with NMDAR encephalitis (Al-Diwani et al. 2019).

However, sGCs have been less thoroughly investigated, and not used as standard immunotherapy in psychiatric disease because of the potential side effects in terms of psychopathological worsening. Alternatives to modulate inflammatory functions in psychiatric disorders are several anti-inflammatory agents, such as cyclooxigenase2-inhibitors, *N*-acetyl-cysteine, or minocycline: a recent meta-analysis reported less pronounced psychotic symptoms when they are applied in addition to antipsychotic treatment, but not when given alone (Jeppesen et al. 2020). Some studies within the spectrum of schizophrenia disorders have investigated their efficacy (Nasib et al. 2020), and others have demonstrated their usefulness in mood disorders (Bodani et al. 1999; Rivera Bonet et al. 2021); animal investigations have revealed recognition memory effects in rodents (Barsegyan et al. 2019).

In recent years, novel disease entities have emerged in psychiatry, such as autoimmune dementia (Flanagan et al. 2010; Banks et al. 2021; Hansen et al. 2021a, b, c; Gibson et al. 2021) or autoimmune psychosis (Pollak et al. 2020). The term autoimmune dementia, a novel entity of neural autoantibody-associated dementia, was coined 10 years ago by Flanagan et al. (2010). This dementia type refers to neural autoantibody-associated dementia that in neuroimaging exhibits a non-neurodegenerative pattern and CSF that suggest inflammation. Furthermore, a positive response to immunotherapy makes the diagnosis probable (Flanagan et al. 2010). Immunosuppressive drugs are required for dementias associated with neural antibodies, such as immunosuppressive agents, like sGCs. Cohort studies suggest that sGCs are the primary therapeutic option in addition to intravenous immunoglobulins (IVIGs) and plasmapheresis, and they may alleviate cognitive dysfunction (Banks et al. 2021; Flanagan et al. 2010). The novel entity of autoantibody-associated psychosis was identified after autoimmune dementia and classified in a recent international consensus (Pollak et al. 2020). Overall, employing sGC in various psychiatric disorders is a promising approach (see Table 1), although the database is small and includes only cases, case series, and cohorts. We briefly highlight in Table 1 promising therapeutic successes for various psychiatric disorders with an autoimmune origin. Further large-scale studies are needed. In other psychiatric disorders, like obsessive–compulsive disorder, an autoimmune origin has been postulated only in recent years, and some evidence has come forth (Endres et al. 2022a, b, d). Relying on trials and clinical experience from autoimmune encephalitis, sGC use is recommended in addition to IVIGs and plasmapheresis as the first therapeutic option (Abboud et al. 2021b). In other psychiatric diseases associated with neural autoantibodies currently treated with psychopharmacologic agents, sGCs are a novel and valuable therapeutic option, i.e., for mood disorders associated with antibodies (Endres et al. 2020; Hansen et al. 2020b) and in those patients exhibiting an isolated or predominant psychiatric manifestation of autoimmune encephalitis (Endres et al. 2022c). This review aims to describe the first preliminary results from early, mostly retrospective pilot studies and to delineate planned studies in the field of autoantibody-associated psychiatric disease.

**Table 1:** Psychiatric disorders that may benefit from systemic glucocorticoid therapy

Psychiatric disorder	Improvement % (*n* response/*n* whole cohort)	References
Autoimmune psychosis	33 (15/46)	Flanagan et al. (2010)
Autoimmune obsessive compulsive disorder	100 (1)	Endres et al. (2022a)
Autoimmune dementia	0 (7)–64 (46/72)	Flanagan et al. (2010); Hansen et al. (2022)
Autoimmune catatonia	100 (1)	Samra et al. (2020)
Autoimmune mood disorder	33 (15/46)	Flanagan et al. (2010)
Psychiatric autoimmune encephalitis	80–93 (21/91–70/91)	Endres et al. (2022c)
Autoimmune based psychiatric syndrome^a^	84 (106/126)	Endres et al. (2020)

^a^The following symptoms could be potentially relevant here: altered consciousness, disorientation, memory impairment, obsessive–compulsive behavior, psychosis, catatonia, mood dysfunction, anxiety, behavioral abnormalities (autism, hyperkinetic), and sleeping dysfunction (Hansen et al. 2020b)

## Methods

As methods in this narrative article, we relied on PubMed to search for articles mentioning the following items: “antibody psychiatry steroid,” “antibody psychiatry synthetic glucocorticoid,” “antibody psychiatry methylprednisolone,” “antibody psychiatry prednisone,” “autoantibody psychiatry steroids,” “autoantibody synthetic glucocorticoids,” “autoantibody psychiatry methylprednisolone,” and “autoantibody psychiatry prednisone” in June 2022. We searched in detail for studies with patients presenting neural antibodies in conjunction with psychiatric symptoms or syndromes and steroids (like methylprednisolone or prednisolone) application. We excluded patients with neurological disorders apart from dementia, internal medicine diseases, such as pancreatitis, autoimmune encephalitis with a mixed neuropsychiatric or no pure psychiatric symptoms, and psychiatric syndromes associated with non-neural antibodies, such as thyroid antibodies. The efficacy of sGCs has been proven in patients with autoimmune encephalitis associated with membrane-surface autoantibodies (Hayden et al. 2021) more than intracellular paraneoplastic antibodies (Hansen et al. 2016). Thus, analogous to sGC effectiveness in patients with neuropsychiatric symptoms and associated neural autoantibodies, it is highly probable that if neural autoantibodies are involved in a disease’s pathogenesis [evident in some neural autoantibodies, such as NMDAR antibodies (Malviya et al. 2017)], sGCs will be effective as well in patients with psychiatric symptoms—although less data are available for that specific indication.

## Results

### Psychiatric disease associated with autoantibodies

Several adult psychiatric-syndrome conditions are associated neural autoantibodies (for review see Hansen et al. 2020b; Pollak et al. 2020; Endres et al. 2022c). The pathophysiological role of these neural autoantibodies is still unclear. Further research is required to clarify whether these autoantibodies are pathogenic, an epiphenomenon, or even protective in some cases. Endres performed a study (Endres et al. 2020) in a huge cohort of patients exhibiting dementia-like and psychotic syndromes as the most prominent psychiatric syndromes associated with neural autoantibodies. 119 of 145 (82%) of those patients received corticosteroids as immunotherapy. Although no information was provided on which type of neural autoantibodies were detected in conjunction with which psychiatric syndromes, 106 of 126 (84%) patients demonstrated a good response to immunotherapy. High-dose immunotherapy (steroid dose above 500 mg/day) in this study was administered more often (60%) than low-dose sGCs (steroid dose under 500 mg/day) (25%), following the latest guidelines for treating autoimmune encephalitis (Abboud et al. 2021a, b) or autoimmune psychosis (Pollak et al. 2020). Moreover, those patients with antibodies against membrane-surface antigens in this study (Endres et al. 2020) received sGCs more often (93%) than those with antibodies against intracellular antigens (67%). A positive response to immunotherapy was detected in 91% of psychiatric patients associated with neural surface autoantibodies, and in 67% of those patients with antibodies against intracellular antigens, confirming results in autoimmune encephalitis demonstrating a stronger response in those patients with cell-surface rather than intracellular autoantibodies. A recent study from Endres et al. (2022c) in patients with psychiatric possible and probable autoimmune encephalitis showed that high-dosage corticosteroids were applied in 57% of all 91 patients, which led to an improvement in 72% of patients. Low-dosage corticosteroids were utilized in 38% of all 91 patients and triggered an improvement in 81%. These study results confirm the usefulness of steroids at a low or high dosage in patients with autoantibody-associated psychiatric disease apparently originating from a possible or probable autoimmune encephalitis in that case series. There is retrospective evidence of case series in autoimmune encephalitis with LGI1 antibodies that steroids (intravenous, oral or both) are even superior to immunoglobulins applied intravenously in functional outcome terms (Rodriguez et al. 2022).

### Neural autoantibody-associated psychiatric disease versus psychiatric disease due to known a physiological condition specified as neural autoantibodies

There is a distinction drawn between psychiatric disorders, and those caused by a known physiological anomaly (organic psychiatric disease). There is evidence that the origin of neural autoantibody-associated psychiatric disease is organic, as in autoimmune encephalitis (Graus et al. 2016) or autoimmune psychosis (Pollak et al. 2020) if a patient exhibits additional CSF indications of a CNS inflammation or a signal increase in brain MRI sequences (FLAIR or T2), as well as neural cell-surface autoantibodies [such as *N*-methyl-D-aspartate receptors (NMDAR) antibodies]. Concerning autoimmune encephalitis following the Graus criteria (Graus et al. 2016), clinicians should differentiate between a possible, probable, or definitive autoimmune encephalitis depending on the extent and grade of CNS inflammation detectable in the brain (unilateral signal anomalies in the temporal lobe in possible, and bilateral signal anomalies in the temporal lobe in probable autoimmune encephalitis). A definitive autoimmune encephalitis is present if IgG autoantibodies are detected in the CSF (Graus et al. 2016). Cell-surface autoantibodies are believed to be pathogenic (Burbelo et al. 2021), whereas autoantibodies against intracellular antigens probably involve cytotoxic T cells (Bien et al. 2012) as their pathogenic mechanism. If an association between neural autoantibodies (like intracellular antibodies) is detected only in serum but not in the CSF with no additional indications of neuronal brain damage or inflammation, no clear autoimmune origin can be postulated. Neural autoantibodies do not per se justify the assumption of an organic basis, as some autoantibodies (like GAD65 autoantibodies) can depict an epiphenomenon; another pathomechanism (such as T-lymphocytes) is suspected of causing autoinflammation (Burton et al. 2010). In these cases, immunotherapy is very controversial, but sometimes also applied as individual experimental therapy, like for some types of atypical dementia (Doss et al. 2014) or autoantibody-associated syndromes, as evident in the Endres et al. (2020) retrospective analysis in which diagnostics sometimes deliver doubtful or unclear indications of an underlying autoimmune basis. Thus, below we discuss examples of patients with organically based psychiatric disease who underwent sGC therapy, or those patients with a psychiatric disease associated with potential signs of inflammation.

### Synthetic glucocorticoids in autoimmune dementia and atypical dementia associated with neural autoantibodies

A positive response to immunotherapy, including intravenous methylprednisolone, was evident in 46 of 72 patients (64%) who underwent this therapy for autoimmune dementia (Flanagan et al. 2010) (Tables 1, 2). Different sGC formulas were applied in this study: intravenous methylprednisolone with different application regimens, and oral prednisone. The responding patients with autoimmune dementia presented autoantibodies against amphiphysin, GAD65, voltage-gated potassium channels, acetylcholine receptors, and calcium channels (Flanagan et al. 2010). Another study described atypical dementia associated with *N*-methyl-d-aspartate receptors (NMDAR) antibodies and partly responsive to polyimmunotherapy, including sGCs (Doss et al. 2014). However, a response to sGCs as first-line immunotherapy was only detected in 50% of patients presenting a membrane-surface autoantibody-mediated cognitive dysfunction (Ariño et al. 2016).

**Table 2:** Synthetic glucocorticoids in psychiatric disease associated with neural autoantibodies

Number of patients	Type of neural autoantibody	Outcome of sGC monotherapy/polytherapy	References
*Dementia*			
57/145	NMDAR, LGI1, CASPR2, AMPAR, GABAR, DPPX, GAD65, ARHGAP26, Ma1/2, Ri, Hu, Yo, AK5, BRSK2	PT: 106/ 126 (84%) whole cohort unclear who many patients with dementia responded to PT	Endres et al. (2020)
2/5	IgANMDAR	PT: 1/2 (50%)	Doss et al. (2014)
72	Amphiphysin, GAD65, VGKC, AchR and CaCh,	PT: 46/72 (64%)	Flanagan et al. (2010)
	LGI1		Ariño et al. (2016)
4/7	IgANMDAR	PT: Improvement	Prüss et al. 2012
1	IgLON5	MT: sGC improvement	Hansen et al. (2020a)
1	Neurexin3alpha	MT: sGC no deterioration	Hansen et al. (2021b)
1	Flotilin 1/2	MT: sGC improvement	Hansen et al. (2021a)
*Psychosis*			
20/72	Amphiphysin, GAD65, VGKC AchR and CaCh	PT: 15/46 (33%) unclear who many patients with psychosis responded to PT	Flanagan et al. (2010)
50/145	NMDAR, LGI1, CASPR2 AMPAR, GABAR, DPPX GAD65, ARHGAP26, Ma1/2, Ri, Hu, Yo, AK5, BRSK2	PT: 106/ 126 (84%) whole cohort unclear how many patients with psychosis responded to PT	Endres et al. (2020)
7	NMDAR	PT: Improvement	Prüss et al. (2012)
*Catatonia*			
1	GABAAR	PT: Improvement	Samra et al. (2020)
*Mood disorder*			
16/145	NMDAR, LGI1, CASPR2 AMPAR, GABAR, DPPX GAD65, ARHGAP26, Ma1/2, Ri, Hu, Yo, AK5, BRSK2	PT: 106/ 126 (84%) whole cohort unclear how many patients with mood disorder responded to PT/ MT	Endres et al. (2020)
20	NMDAR		Restrepo-Martínez et al. (2020)
1	IgM NMDAR	MT: Improvement	Choe et al. (2013)
25/ 72	Amphiphysin, GAD65, VGKC AchR and CaCh	PT: 15/46 (33%) unclear how many patients with mood disorder responded to PT	Flanagan et al. (2010)

*AcHR* acetylcholine receptor, *ARHGAP26* Rho GTPase-activating protein 26, *BRSK2* brain selective Kinase 2, *CaCh* calcium channel, *CASPR2* contactin-associated protein-like 2, *GABAAR* gamma aminobutyric acid A receptor, *GAD65* glutamic acid decarboxylase 65, *LGI1* leucine-rich glioma-inactivated protein 1, *NMDAR* *N*-methyl-d-aspartate receptor, *PT* polytherapy, *VGKC* voltage-gated potassium channel, *MT* monotherapy

### Synthetic glucocorticoids in psychotic disorders and psychotic disorders caused by neural antibodies

The Flanagan et al. (2010) study of autoimmune dementia was characterized by psychotic symptoms, including hallucinations in 20 of 72 patients. 15 of 46 (33%) of their patients responded, whereas 5/26 (19%) did not respond to immunotherapy, including steroids (Tables 1, 2). A study is being planned will investigate prednisolone versus placebo in patients suffering a recent-onset psychotic disorder within the previous seven years, including 90 subjects with schizophrenia, schizoaffective disorder, or schizophreniform disorder (Nasib et al. 2020). The primary outcome criteria are a lower positive and negative syndrome scale (PANSS) score encompassing psychotic symptoms, as well as various immunological markers after a 40 mg/day dose of prednisolone slowly reduced over a 6-week time period. The advantage of low-dose prednisone will be made obvious through the lower risk for side effects (i.e., inducing psychiatric symptoms) as evident in the Fardet et al. (2012) study.

### Synthetic glucocorticoids for catatonia caused by neural autoantibodies

A further psychiatric disease entity is catatonia—often considered an aspect of schizophrenia—but nowadays also classified as an independent psychiatric disease entity (Gazdag et al. 2017). Furthermore, catatonia might appear as a symptomatic manifestation of autoimmune encephalitis, as in NMDAR encephalitis (Al-Diwani et al. 2019). Corticosteroids combined with other immunosuppressive agents alleviated catatonia in patients with gamma amino butyric acid A (GABAA)-receptor antibody catatonia (Samra et al. 2020) (Tables 1, 2).

### Synthetic glucocorticoids in mood disorders, and those caused by neural autoantibodies

In patients with autoimmune dementia and additional depressed mood as a probable symptom manifestation of autoimmune dementia beyond cognitive dysfunction, steroids proved to be beneficial in 15/46 (33%) of patients (Table 2), whereas they revealed no effect in 10/26 (38%) of patients. However, it is noted that corticosteroids were not applied exclusively, but in combination with other immunotherapies (Flanagan et al. 2010). In a manic syndrome associated with NMDAR antibodies, steroids led to a remission of symptoms (Restrepo-Martínez et al. 2020).

### Synthetic glucocorticoids: usefulness in psychotic and mood disorders involving anti-thyroid autoimmunity in conjunction with neural autoantibodies

A large recent chart review study by Endres et al. (2021) showed that neuronal autoantibodies occur much more often in patients with schizophreniform or affective disorders also associated with anti-thyroid antibodies than those without anti-thyroid antibodies. The authors postulated an impact from the thyroid antibodies on vascular muscle cells regulating the blood–brain barrier function; they speculated that a worsening blood–brain barrier disturbance via anti-thyroid antibodies predisposes the brain for autoimmune processes leading to more prevalent neuronal autoantibodies in these patients. It would thus make sense to administer sGCs in such patients also, although sometimes the autoimmune basis is unclear. However, the sole occurrence of anti-thyroid antibodies does not justify the diagnosis of an autoimmune encephalopathy that could undergo sGC treatment, as other diseases might be being mimicked (Valencia-Sanchez et al. 2021).

### Mechanisms of synthetic glucocorticoid efficacy in neural autoantibody-associated psychiatric disease

**Fig. 1:**
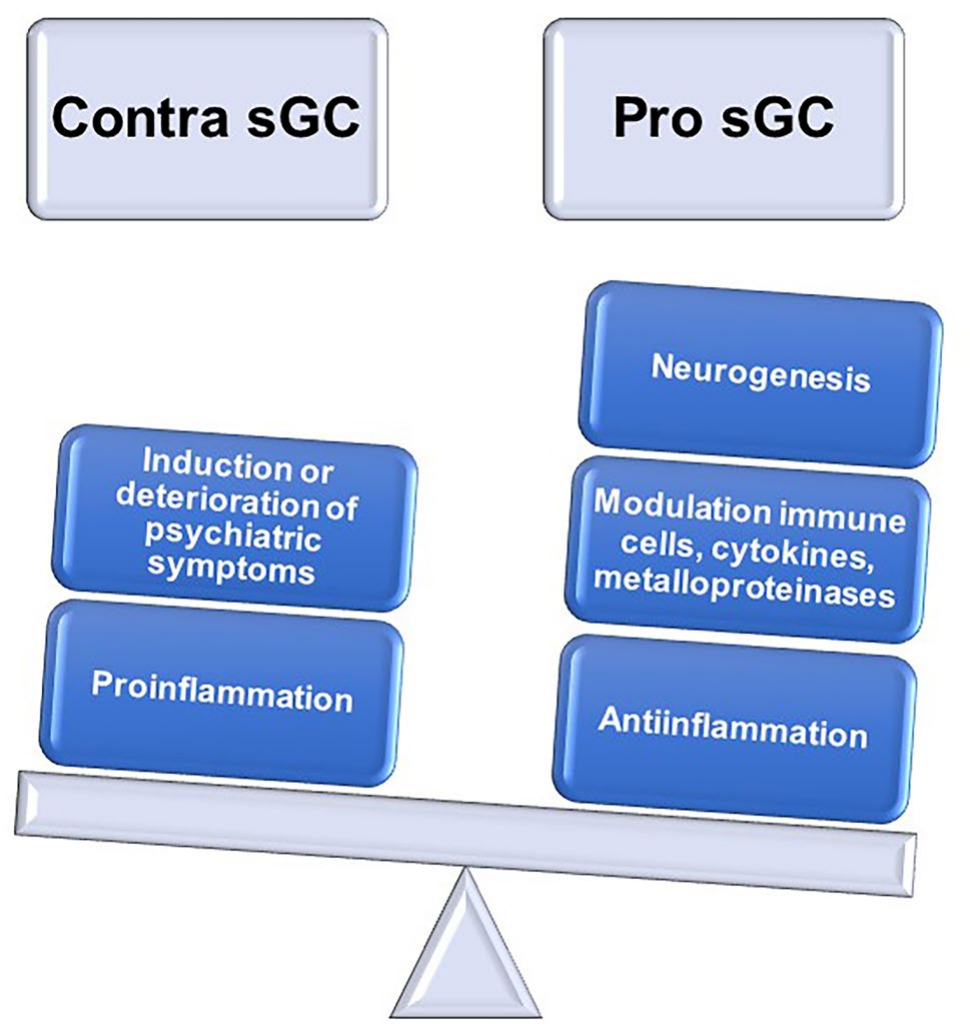
Considering the advantages and disadvantages of synthetic glucocorticoids in psychiatric disease associated with neural autoantibodies—a schematic overview. The advantages outweigh the disadvantages regarding sGC use for neural autoantibody-associated psychiatric disease. *sGCs* synthetic glucocorticoids

Different sGC mechanisms with a major impact on neural autoantibody-associated psychiatric disease should be mentioned. First, sGCs can enhance emotional memory by increasing hippocampal activity during the encoding of emotional stimuli (Kukolja et al. 2011) with a neuro-enhancing effect. This type of therapy may especially help patients suffering hippocampus-based memory deficits caused by a CNS inflammation within the hippocampal system. Second, GCs can induce neurogenesis as a neuroprotective effect through, for example, the induction and upregulation of a glucocorticoid-induced leucine zipper protein that furthermore downregulates peroxisome proliferator-activated receptors and adipogenic transcription factor 2—both relevant for neurogenesis (Srinivasan Lahiri 2016). Third, GCs can modulate cellular immune responses, such as the T cells (Liberman et al. 2018), critical for neuronal survival. More specifically, GC can help in conditions of autoimmunity as they can reduce inflammation by influencing regulatory T cells and by promoting the traffic from T cells back to their bone marrow and lymphoid tissues via various mechanisms (such as inducing chemokines or metalloproteinases) (Besedovsky et al. 2014; Fischer et al. 2013; Liberman et al. 2018). In addition, antigen presenting cells, such as dendritic cells, are also influenced by GCs (de Jong et al. 1999). This is relevant, as these dendritic cells are important in producing neural autoantibodies. Fourth, a GC on the glucocorticoid receptor can induce genomic changes by inducing altered pro- or anti-inflammatory gene expression (Thibaut 2019). Thus, sGCs have many beneficial effects that are superior in balancing the pros and cons—although their potential side effects cannot be ignored (Fig. 1). Nevertheless, our review describes not all sGC factors, and only some thereof are presented of in detail. The complex role played by sGCs within the immune system is not fully considered in our review. A perhaps neglected yet important topic is also the role sGCs play in the development of T cells and their various functions (for a review, see Taves and Ashwell 2021). In addition, the role of sGCs in the interaction between the immune system and endocrine system, which is complex (Cain and Cidlowski 2017), is not addressed here, but should be considered especially when autoantibodies to thyroid antigens are detected in patients with psychiatric symptoms. The other novel avenue to consider when considering carefully the sGC mechanism is their potentially regenerative role, as recently shown in the interaction between regulatory T cells and hair follicle stem cells, as in the study by Liu et al (2022). Thus, the complex role of sGCs is an understudied topic that should be explored in future studies addressing their mechanisms in psychiatric autoimmune diseases.

### Steroid psychosis as example of corticosteroid-induced psychiatric disorder

One factor limiting sGC therapy for autoantibody-associated psychiatric disease is its potential to induce a psychosis or mood disorder. Steroid-induced psychosis or mania are severe adverse effect of steroids. These effects are dose dependent. Nevertheless, there are several strategies to relieve such psychiatric symptoms, i.e., by reducing the steroid dose of or even completely stopping the drug, as recommended recently by Huynh and Reinert (2021). Moreover, inpatient treatment with the possibility to closely monitor psychopathological worsening may be another option to increase patient safety. We recommend that each patient be evaluated for such a risk of triggering or exacerbating psychiatric symptoms, such as psychosis, e.g., in patients with severe psychotic symptoms. In such patients, we do not recommend sGC use; switching to IVIGs might be a good alternative. We therefore strongly recommend being aware of the possible side effect of exacerbating or triggering psychiatric symptoms, although this is usually manageable and often worth trying with sGCs as a long period of no treatment for psychiatric autoimmune encephalitis may lead to a permanently worse clinical outcome.

### Synthetic glucocorticoid formulation for neural autoantibody-associated psychiatric disease

The application of sGCs in psychiatric disease is often complicated by side effects, as they can intensify psychiatric symptoms. However, a study by Fardet et al. (2012) showed that these side effects are dose-dependent cautious with high-dose steroids and that the dosage itself might be more likely to cause neuropsychiatric symptoms. There is no need for a novel formula for sGCs in neural autoantibody-associated psychiatric syndromes as the autoantibody spectrum shows substantial overlap with autoimmune encephalitis. Thus, for patients with neural autoantibody-associated syndromes, sGCs should be administered according to the guidelines proposed for autoimmune encephalitis (Abboud et al. 2021a, b; Graus et al. 2016). sGCs, when applied as an intravenous high-dosage sGC (1 g/day)-like methylprednisolone over a 3–7-day period repeated monthly for six months, are therefore recommended to alleviate autoantibody-associated psychiatric syndromes. High doses of corticosteroids carry a much lower risk of long-term sequelae such as cataract or osteoporosis. The clinician should aim to weigh carefully the pros (avoiding the long-term effects of low-dose steroids) and cons (inducing psychiatric symptoms) of sGCs in each patient. A factor in support of tolerating the induction of short-term psychiatric symptoms is that they are much easier to treat than are the long-term, irreversible effects of low-dose steroids. We this recommend prioritizing high-dosage sGCs for patients with autoantibody-associated psychiatric syndromes.

## Discussion

This paper has presented initial, preliminary data from the growing field of neural autoantibody-associated psychiatric disease regarding the usage of and beneficial effects of sGCs. The advantages of steroids are particularly obvious when a patient exhibits strong evidence of an autoimmune encephalitis accompanied by isolated or predominant psychiatric symptoms, analogous to standard treatment for autoimmune encephalitis (Abboud et al. 2021a, b). Overall, the benefits of sGCs (i.e., rapidly alleviated psychiatric symptoms or disease resolution) outweigh their negative effects and other aspects (e.g., the absence of randomized, placebo-controlled trials and the minimal risk of inducing psychiatric symptoms). However, further large-scale studies are needed to investigate sGCs’ efficacy in alleviating neural autoantibody-associated psychiatric disease. There is ample evidence that patients’ sensitivity to sGCs varies widely, as some are resistant to them [their resistance being caused by downregulated glucocorticoid receptors or states of phosphorylation (Ramamoorthy and Cidlowski 2016)]. Overall, our data suggest that sGCs are a valuable and promising option in treating several autoimmune-mediated psychiatric disorders (Tables 1, 2). We have formed our opinion according to specific information on the benefits, efficacy, and safety of sGC use in patients with autoimmune-mediated psychiatric disorders. For clinical administration, a balance must always be struck between the potential benefits, which as described can be quite diverse, and potential harm by exacerbating psychiatric symptoms. Such balancing of interests (Fig. 1) is always recommended and should not be disregarded despite the obvious autoimmune-mediated cause. All individual potential effects and side effects are certainly not to be considered, since the immunological role of glucocorticoids is extremely complex and multifaceted. When balancing such interests, besides the clinical advantages, we also need to keep in mind also fundamental aspects, such as the effect of sGC on neurogenesis, other immune cells, such as T cells, and generally anti-inflammatory aspects (Fig. 1). However, from a theoretical point of view, the proinflammatory effects of glucocorticoids (Fig. 1) argue against their use, in addition to safety aspects.

### Limitations

Several limitations have to be addressed: First, our expert opinion review is narrative and not systematic; thus, we did not involve the German Clinical Trials Register, or ClinicalTrials.gov from the U.S. National Library of Medicine and World Organisation of Health clinical trials for planned studies and other databases, like Excerpta Medica Database, Psychological information database, and Cochrane Database of Systematic Reviews—all of which should be consulted in a future meta-analysis. Furthermore, the heterogeneity of classification systems, i.e., that of Graus et al. (2016) between autoimmune psychosis according to Pollak et al. (2020), or autoimmune dementia according to Flanagan et al. (2010), and autoimmune- based psychiatric syndromes (Hansen et al. 2020a, b), make it problematic to make general recommendations for the use of sGCs.

### Conclusions

We suggest that trials be conducted targeting individual disease entities, such as specific psychotic disorders or dementias. Neural autoantibody-associated psychiatric disease represents a novel indication for applying sGCs and thus the repositioning of an old drug, like sGC. Further attention must be paid in future trials on the occurrence of adverse events, on psychiatric symptoms aggravated by sGCs, and on how such side effects might be prevented or minimized. Based on the knowledge of autoimmune-related psychiatric disorders described and summarized in our results, we conclude that sGCs are a valuable therapeutic option when neural autoantibodies are detected in psychiatric patients in conjunction with IgG antibodies in the CSF or with other clinical and laboratory parameters suggesting CNS inflammation. Although our review is narrative and the evidence level presented is low to moderate (because case series and cohorts are presented as our basis for conclusions), our review data point to a new direction in psychiatry that has emerged in recent years and should be pursued further. However, the use of sGCs must be seriously reconsidered when potent alternative immunotherapeutic drugs such as IVIGs and plasmapheresis are available, in case side effects such as exacerbation of psychiatric symptoms should occur. In every case, a critical risk–benefit evaluation should be made especially in cases with isolated psychiatric syndromes and lacking neural autoantibody detection in CSF.

## Data Availability

No novel data were build or analyzed in this investigation. Data sharing is thus not applicable to this article.
